# Life Cycle Stage-Specific Accessibility of Leishmania donovani Chromatin at Transcription Start Regions

**DOI:** 10.1128/mSystems.00628-21

**Published:** 2021-07-20

**Authors:** Janne Grünebast, Stephan Lorenzen, Julia Zummack, Joachim Clos

**Affiliations:** a Leishmaniasis Group, Bernhard Nocht Institute for Tropical Medicine, Hamburg, Germany; b Department of Tropical Epidemiology, Bernhard Nocht Institute for Tropical Medicine, Hamburg, Germany; University of California San Diego

**Keywords:** nucleosomes, micrococcal nuclease, radicicol, HSP90, strand switch regions

## Abstract

Leishmania donovani is a parasitic protist that causes the lethal Kala-azar fever in India and East Africa. Gene expression in *Leishmania* is regulated by gene copy number variation and inducible translation while RNA synthesis initiates at a small number of sites per chromosome and proceeds through polycistronic transcription units, precluding a gene-specific regulation (C. Clayton and M. Shapira, Mol Biochem Parasitol 156:93–101, 2007, https://doi.org/10.1016/j.molbiopara.2007.07.007). Here, we analyze the dynamics of chromatin structure in both life cycle stages of the parasite and find evidence for an additional, epigenetic gene regulation pathway in this early branching eukaryote. The assay for transposase-accessible chromatin using sequencing (ATAC-seq) analysis (J. D. Buenrostro, P. G. Giresi, L. C. Zaba, H. Y. Chang, and W. J. Greenleaf, Nat Methods 10:1213–1218, 2013, https://doi.org/10.1038/nmeth.2688) predominantly shows euchromatin at transcription start regions in fast-growing promastigotes, but mostly heterochromatin in the slowly proliferating amastigotes, the mammalian stage, reflecting a previously shown increase of histone synthesis in the latter stage.

**IMPORTANCE** *Leishmania* parasites are important pathogens with a global impact and cause poverty-related illness and death. They are devoid of classic *cis*- and *trans*-acting transcription regulators but use regulated translation and gene copy number variations to adapt to hosts and environments. In this work, we show that transcription start regions present as open euchromatin in fast-growing insect stages but as less-accessible heterochromatin in the slowly proliferating amastigote stage, indicating an epigenetic control of gene accessibility in this early branching eukaryotic pathogen. This finding should stimulate renewed interest in the control of RNA synthesis in *Leishmania* and related parasites.

## INTRODUCTION

The nuclear DNA of eukaryotes is known to be highly and reversibly organized into DNA-protein assemblies known as chromatin, which allows compacting, stabilization, and regulated access to chromosomes or parts thereof. The smallest unit of chromatin is the nucleosome, which consists of an octamer of four basic proteins, histones H2A, H2B, H3, and H4, around which a short length of DNA, 146 bp, is wrapped (1, 2). This 10-nm-wide fiber, also called beads-on-a-string, forms the euchromatin, which is accessible to regulatory protein complexes, namely, replication and transcription complexes (3). Facilitated by the linker histone H1, the 10-nm fiber can be condensed into 30-nm fibers, which are thought to form the heterochromatin, the less accessible form of nuclear DNA (4, 5).

The local density of chromatin and the exact positioning of nucleosomes in or near protein-coding genes are part of what constitutes epigenetic gene expression regulation, modulating the access to *cis*-regulatory DNA sequences (6, 7). This allows eukaryotic cells to compact chromatin that is not actively transcribed or replicated, which is important given the size and complexity of eukaryotic genes, and of eukaryotic genomes in general, and their reliance on regulated transcription.

The protist order Trypanosomatida comprises early-branching eukaryotes and includes important parasitic pathogens of animals and humans, such as the causative agents of sleeping sickness (Trypanosoma brucei ssp.), Chagas disease (Trypanosoma cruzi), and also cutaneous leishmaniasis (e.g., Leishmania major) and visceral leishmaniasis (Leishmania donovani, Leishmania infantum). These so-called trypanosomatidic infections are among the most important of the neglected tropical diseases, causing widespread, poverty-related human morbidity and mortality in tropical and subtropical regions around the world (https://www.who.int/data/gho/data/themes/topics/gho-ntd-leishmaniasis) (8).

They are distinct from the other eukaryotes in that they lack gene-specific transcription regulation, most known transcription regulator proteins, and canonical gene promoters (9), instead relying on posttranscriptional mechanisms, such as modulated RNA stability, RNA processing activity, translation efficiency, and reversible gene and chromosome amplification (10). Their genomes are 2 orders of magnitude smaller (∼3.2 × 10^7^ bp) than those of mammals (∼ 3 × 10^9^ bp), encoding ∼8,200 protein-coding genes, which for the most part lack introns (11). An interesting feature is the multicistronic, continuous transcription of large head-to-tail arrays of functionally nonrelated genes. These multicistronic transcription units are located on alternating strands of the chromosomes (10, 12) and are flanked by so-called convergent or divergent strand switch regions (SSRs), with the latter shown to be starting points of multicistronic transcription (13, 14). Early evidence also suggests that the predominant form of chromatin in trypanosomatids is the euchromatin, i.e., the 10-nm beads-on-a-string structure, while 30-nm fibers have not been observed (15).

The *Leishmania* spp. are parasites of phlebotomine sandflies (*Phlebotomus* spp., *Lutzomyia* spp.) and of antigen-presenting cells (APCs), e.g., macrophages, in their mammalian hosts. In the sandflies, they exist as elongated, flagellated “promastigotes,” which are highly proliferative. Upon transmission into a mammal, they are phagocytosed by neutrophil cells, dendritic cells, and macrophages. Triggered by mammalian tissue temperature and the acidic milieu inside phagosomes, they undergo a reversible stage conversion to the ovoid, aflagellated “amastigotes” that proliferate within the host cells at moderate to minimal growth rates (16, 17). Ultimately, the macrophage host cell is destroyed, and the free amastigotes are phagocytosed by other APCs, continuing their intracellular persistence. The destruction of host cells and the concomitant release of parasite antigen triggers the immune pathologies that are characteristic for the infecting *Leishmania* species, cutaneous lesions or generalized pathology of visceral organs (18). The latter form has a lethal outcome if not treated efficiently.

Culture-adapted Leishmania donovani usually can be triggered to undergo promastigote-to-amastigote stage conversion when subjected to elevated temperature (37°C) and acidic pH (pH 5.5) (19), which allows researchers to study molecular events during cellular differentiation (20). A similar differentiation is triggered when L. donovani promastigotes are treated with inhibitors of the major chaperone protein HSP90, such as geldanamycin or radicicol (RAD) (21, 22). This effect is abrogated when L. donovani expresses the mutant HSP90rr, which carries a single leucine to isoleucine amino acid exchange in its ATP binding site and confers resistance to RAD-induced growth inhibition and cellular differentiation (22).

While *Leishmania* can adapt to environmental challenges by chromosomal aneuploidy and extrachromosomal and intrachromosomal gene amplification, thereby altering relevant gene copy numbers (23–25), short-term responses to environmental stimuli appear to be regulated at the posttranscriptional level. This includes the triggers for stage conversion. A recent genome-wide transcriptome sequencing (RNA-seq)/ribosome profiling analysis revealed that (i) inducible changes of RNA abundance and protein synthesis correlate poorly and (ii) HSP90 inhibitor-triggered differentiation causes an upregulation of the synthesis of a large group of amastigote-specific proteins (26). In addition to those, an increased synthesis of histone proteins was also observed, which matched an earlier proteome study (20). This raises the question of whether increased histone levels may impact the chromatin structure of L. donovani during promastigote-to-amastigote differentiation.

In this paper, we show that nuclear DNA in axenic amastigotes and in RAD-induced cells is less sensitive to micrococcal nuclease degradation. Furthermore, an assay for transposase-accessible chromatin (ATAC) analysis showed a strong reduction of accessibility in divergent strand switch regions and 5′-telomeric regions in the axenic amastigote, indicating a switch from euchromatin to heterochromatin at transcription start sites and a possible epigenetic impact on stage-specific RNA synthesis.

## RESULTS

### Heat shock reduces MNase accessibility of L. donovani chromatin.

**FIG 1:**
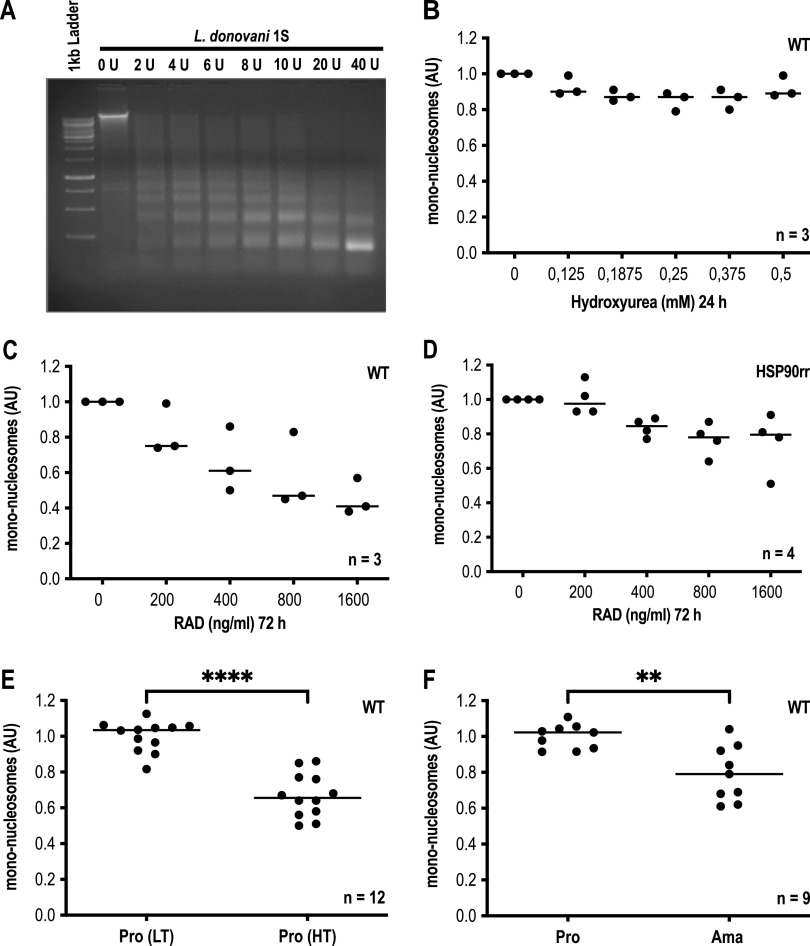
Micrococcal nuclease digest of L. donovani nuclear DNA. A total of 5 × 10^7^ cells per sample were permeabilized and treated with MNase for 5 min at room temperature. The reaction was stopped, and samples were incubated for 2 h at 37°C with proteinase K. DNA was precipitated with ethanol, and 3 μg of DNA was analyzed on a 1.5% agarose gel. The intensity of mononucleosome-derived DNA bands was quantified using Image J software and normalized to control. (A) MNase dose testing and impact on the intensity of mononucleosome-derived DNA bands. (B) 40 units/ml MNase. (C to F) 80 units/ml MNase. (B) Treatment of L. donovani promastigotes with hydroxyurea for 24 h at 25°C. (C) Treatment of L. donovani promastigotes with radicicol (RAD) at various concentrations for 72 h at 25°C. (D) The same as in panel C, but with L. donovani (HSP90rr) promastigotes. (E) L. donovani promastigotes at 37°C for 72 h. (F) Axenic L. donovani amastigotes at day 4 of differentiation. Significance for panels E and F was tested using the ratio-paired *t* test. ****, *P* ≤ 0.0001; **, *P* ≤ 0.01; low temperature (LT) = 25°C; high temperature (HT) = 37°C.

We first examined whether environmental signals have an impact on the overall density of chromatin in L. donovani. For this, we performed micrococcal nuclease (MNase) digests (1) on permeabilized cells. Figure 1A shows the MNase dose-dependent digest of promastigote chromatin at fixed temperature and reaction time. The agarose gel image shows MNase fragments in 200-bp increments down to the smallest, 200-bp products that represent the protected DNA of single nucleosomes. For the following experiments, we chose 40 to 80 units of MNase and used ImageJ analysis to quantify the fraction of DNA in the 200-bp band as a measure of chromatin accessibility.

First, we tested whether a hydroxyurea-mediated growth arrest in the G_1_ cell cycle phase alters the general MNase accessibility of promastigotes grown at 25°C and pH 7.4 (Fig. 1B). No significant differences could be observed at up to 80% inhibitory concentration (IC_80_) of the compound, indicating that mere growth arrest has no impact on the chromatin density.

We next tested the impact of the HSP90 inhibitor RAD on L. donovani promastigotes at 25°C/pH 7.4 (Fig. 1C). The abundance of the 200-bp band dropped by ∼60% at the highest RAD concentration, indicating that the observed increase of histone synthesis in RAD-treated leishmania (26) indeed reduces chromatin accessibility. This was further confirmed when we performed the same experiment using L. donovani cells that expressed the HSP90rr variant, which confers RAD resistance (22). Here, the effect on chromatin accessibility was clearly reduced (Fig. 1D). We conclude that RAD-mediated HSP90 inhibition causes not only increased histone synthesis but also changes to the chromatin accessibility.

Since treatment of L. donovani with HSP90 inhibitors is known to mimic exposure to heat shock (21), we also tested the impact of a 72-h exposure to 37°C (Fig. 1E). We indeed observed a 40% reduction of the relative abundance of the 200-bp band. This effect was also seen when we induced axenic-stage conversion from promastigotes to amastigotes by incubation for 96 h at 37°C/pH 5.5 (Fig. 1F). Again, we saw an increased protection against MNase digest.

We therefore conclude that stimuli that trigger parts of the stage conversion pathways, such as elevated temperature, acidic pH, and HSP90 inhibition, reduce the accessibility of nuclear DNA for an endonuclease, suggesting an alteration of chromatin structure.

### ATAC-seq analysis of L. donovani chromatin accessibility.

**FIG 2:**
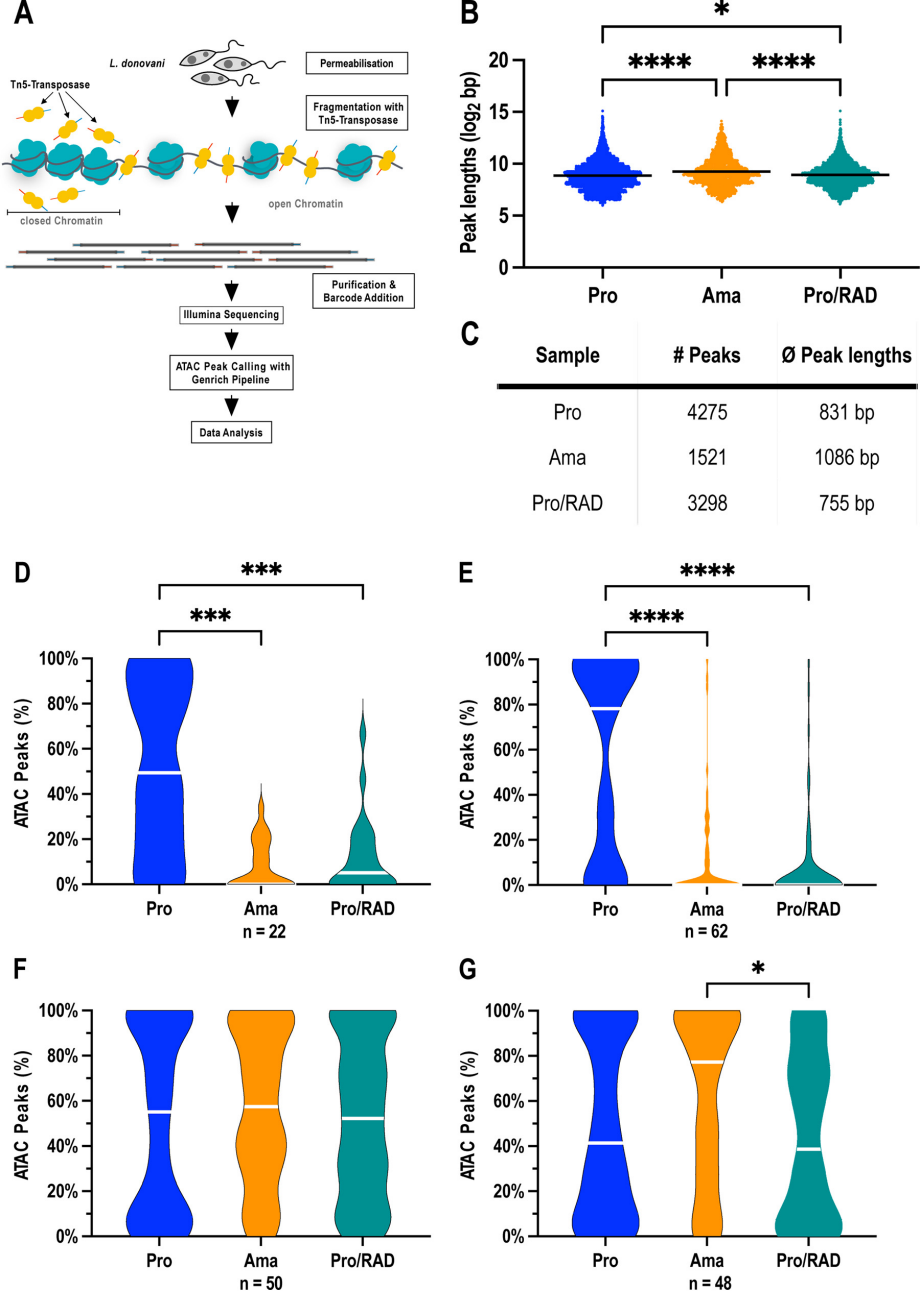
ATAC-seq analysis. (A) Schematic drawing of ATAC-seq analysis in L. donovani. Isolated chromatin was treated with Tn*5* transposase for digest and adapter ligation followed by Illumina next-generation sequencing. Reads were subjected to peak calling using the Genrich pipeline and subsequent analysis steps. (B) Peak lengths in promastigotes (Pro, blue), axenic amastigotes (Ama, orange), and RAD-treated promastigotes (Pro/RAD, green). Each dot represents one peak. Significance was tested using the Kruskal-Wallis test; ****, *P* ≤ 0.0001; *, *P* ≤ 0.05; *n* = 2. (C) Table showing the number of peaks and the average peak lengths. (D to G) Open chromatin at potential transcription origin and termination regions. ATAC peak coverages for promastigotes (Pro, blue), axenic amastigotes (Ama, orange), and RAD-treated promastigotes (Pro/RAD, green) were measured at 5′ telomeres (D), divergent SSRs (E), 3′ telomeres (F), and convergent SSRs (G). Significance was tested using the Kruskal-Wallis test; ****, *P* ≤ 0.0001; ***, *P* ≤ 0.001; *, *P* ≤ 0.05. Data were pooled from 2 biological samples; *n*, number of analyzed regions.

We next sought to test whether the induced changes to the chromatin structure were general or restricted to specific regions of the genome. This can be achieved by an endonucleolytic digest of nuclear DNA followed by next-generation sequencing to pinpoint nuclease-accessible euchromatin within the genome. Figure 2A shows a schematic overview of such a strategy, the assay for transposase-accessible chromatin using sequencing (ATAC-seq) (27). A permeabilized cell population is subjected to fragmentation using the Tn*5* transposase, which, in addition to fragmentation, simultaneously ligates barcode primers to the fragments. The fragment library is then subjected to Illumina sequencing, and the starts of the individual reads identify the positions of Tn*5*-accessible DNA. The ATAC peaks are then identified and quantified relative to Tn*5* fragmentation of pure genomic DNA (https://github.com/jsh58/Genrich). The ATAC-seq read alignment peaks represent areas that are accessible to the Tn*5* transposase and are commonly associated with open chromatin.

We applied ATAC-seq to the following three different cell types, each in duplicate: (i) logarithmically growing promastigote cells, (ii) axenic amastigotes, and (iii) promastigotes treated with RAD (IC_80_). In addition, purified genomic DNA of L. donovani (50 ng and 200 ng) was also treated with Tn*5* transposase as control. Table S1 in the supplemental material shows the yields of sequence reads before and after the trimming of barcodes, and the percentage of reads that could be aligned to the L. donovani reference genome.

10.1128/mSystems.00628-21.1 TABLE S1 ATAC-seq output. Two biological replicates each were used for sequencing of promastigotes (Pro), amastigotes (Ama), RAD-treated promastigotes (Pro/RAD), and genomic DNA controls. Numbers of total reads, number of reads after adapter trimming, and percentage of reads aligned to the L. donovani reference genome are listed. Download Table S1, PDF file, 0.06 MB. Copyright © 2021 Grünebast et al. 2021 Grünebast et al. https://creativecommons.org/licenses/by/4.0/ This content is distributed under the terms of the Creative Commons Attribution 4.0 International license.

We performed a global analysis of peak numbers and lengths (Fig. 2B; see also Fig. S1 in the supplemental material) by calculating peak lengths from the output of Genrich, which shows peak coordinates. This already showed significant differences between the cell types. Compared to promastigotes and RAD-treated promastigotes, axenic amastigotes showed longer (*P* ≤ 0.0001) (Fig. 2B) but fewer (Fig. 2C) peaks. This result is in keeping with the observed reduced nuclease accessibility of amastigote chromatin (Fig. 1F) but not with the MNase digest results of RAD-treated promastigotes (Fig. 1C).

10.1128/mSystems.00628-21.4 FIG S1 Frequency distribution of peak lengths. Peak lengths derived from Genrich peak calling were plotted against their relative frequencies for promastigotes (Pro, blue), axenic amastigotes (Ama, orange), and RAD-treated promastigotes (Pro/RAD, green). Download FIG S1, PDF file, 0.05 MB. Copyright © 2021 Grünebast et al. 2021 Grünebast et al. https://creativecommons.org/licenses/by/4.0/ This content is distributed under the terms of the Creative Commons Attribution 4.0 International license.

### Distribution of euchromatin in L. donovani.

In the next step, we looked at the distribution of ATAC peaks between different regions of chromosomes, namely, telomere regions upstream (5′ telomeres) of polycistronic transcription units (PTUs), telomere regions downstream (3′ telomeres) of PTUs, divergent strand switch regions (dSSRs), convergent SSRs (cSSRs), PTUs, protein-coding sequences (CDSs), and intergenic regions. The results are shown in Fig. S2 in the supplemental material. ATAC peaks in promastigotes show an overproportioned frequency in 5′ telomeric regions and in dSSRs, which are associated with transcription initiation, and an underproportioned representation in 3′ telomers and cSSRs, which are considered transcription termination regions. Their frequency in PTUs, CDSs, and intergenic regions reflects the overall genomic frequency.

10.1128/mSystems.00628-21.5 FIG S2 Distribution of open chromatin across the L. donovani genome. Coverage of ATAC peaks is indicated for promastigotes (Pro, blue), axenic amastigotes (Ama, orange), and RAD-treated promastigotes (Pro/RAD, green) at 5′- and 3′-telomeres, divergent (dSSRs) or convergent strand switch regions (cSSRs), polycistronic transcription units (PTUs), coding sequences (CDSs), intergenic regions within PTUs, and total chromosomes. Data were pooled from 2 biological samples. Download FIG S2, PDF file, 0.06 MB. Copyright © 2021 Grünebast et al. 2021 Grünebast et al. https://creativecommons.org/licenses/by/4.0/ This content is distributed under the terms of the Creative Commons Attribution 4.0 International license.

In addition, we analyzed ATAC peak coverage in tRNA- and rRNA-coding genes (see Tables S2 and S3 in the supplemental material). Our analysis showed them to be universally accessible to Tn*5* transposase, in keeping with earlier analyses (28, 29).

10.1128/mSystems.00628-21.2 TABLE S2 ATAC peak coverage at tRNA genes. Download Table S2, PDF file, 0.1 MB. Copyright © 2021 Grünebast et al. 2021 Grünebast et al. https://creativecommons.org/licenses/by/4.0/ This content is distributed under the terms of the Creative Commons Attribution 4.0 International license.

10.1128/mSystems.00628-21.3 TABLE S3 ATAC peak coverage at rRNA genes. Download Table S3, PDF file, 0.05 MB. Copyright © 2021 Grünebast et al. 2021 Grünebast et al. https://creativecommons.org/licenses/by/4.0/ This content is distributed under the terms of the Creative Commons Attribution 4.0 International license.

### Promastigote-specific open chromatin at dSSRs.

When we analyzed the ATAC peak coverage of 5′ telomeres and dSSRs, the differences became even clearer. For the 22 identified 5′ telomeres analyzed, the median peak coverage was 50% (Fig. 2D), with coverage in amastigote and Pro/RAD samples below 10%. In the 62 identified dSSRs (Fig. 2E), this was even more pronounced. In promastigote samples, median ATAC peak coverage was ∼75%, while it was close to 0% for amastigotes and Pro/RAD cells. For the 50 identified 3′ telomeres, the median ATAC peak coverage was slightly above 50% for all samples, although the polarity between high and low coverages was stronger for the promastigote samples (Fig. 2F). The 48 identified cSSRs showed a different picture. Here, amastigote chromatin showed an increased median ATAC peak coverage (∼75%) compared with that of promastigotes (∼40%) and Pro/RAD cells (∼35%) (Fig. 2G). This indicates that in promastigotes, open chromatin dominates in regions associated with transcription initiation, namely, 5′ telomeres and dSSRs. These findings can be ascertained by visual inspection of ATAC peak patterns relative to transcription direction in Fig. S3 in the supplemental material.

10.1128/mSystems.00628-21.6 FIG S3 Genrich peak calling for all 36 chromosomes in L. donovani. Peak calling was performed for two biological repeats and is depicted for promastigotes (Pro, blue), axenic amastigotes (Ama, orange), and RAD-treated promastigotes (Pro/RAD, green). Coding sequences (CDS) in two different orientations are indicated with black dots (forward direction) and red dots (reverse direction). Download FIG S3, PDF file, 0.8 MB. Copyright © 2021 Grünebast et al. 2021 Grünebast et al. https://creativecommons.org/licenses/by/4.0/ This content is distributed under the terms of the Creative Commons Attribution 4.0 International license.

### Euchromatin to heterochromatin switch immediately upstream of PTUs.

**FIG 3:**
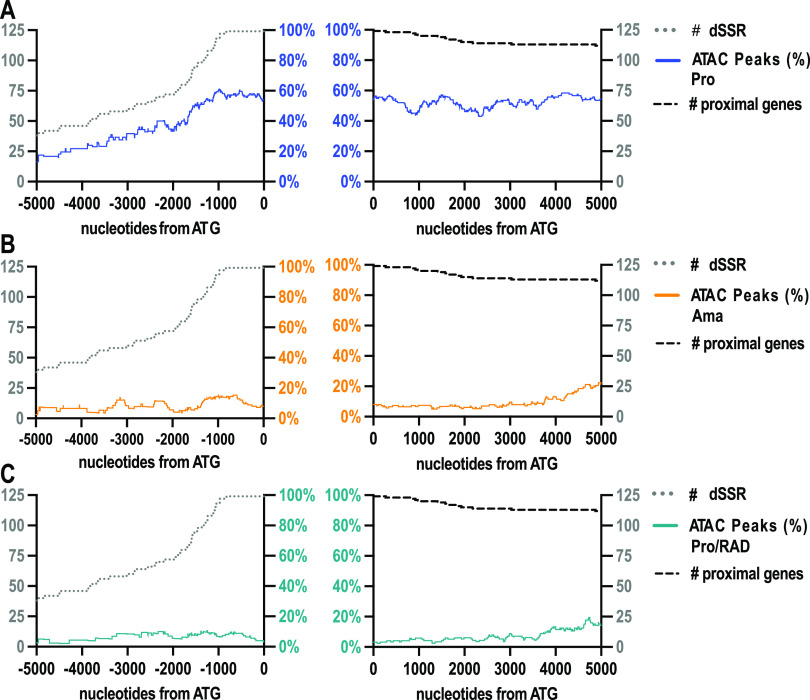
ATAC peaks observed 5′ and 3′ from SSR-proximal CDS start sites. ATAC peak coverage in single-nucleotide resolution upstream (−5,000 bp to 1 bp) and downstream (+1 to +5,000 bp) of CDS start. Coverage is normalized to the number of analyzed divergent (d) SSRs and the number of proximal genes, respectively, for promastigotes (Pro, blue) (A), axenic amastigotes (Ama, orange) (B), and RAD-treated promastigotes (Pro/RAD, green) (C).

To localize euchromatin within the dSSRs, we mapped ATAC peaks from position −5,000 to +5,000, relative to the start of the dSSR-proximal CDS and normalized them against the number of dSSRs. The results are shown in Fig. 3 (for 5′-telomeric start sites, see Fig. S4 in the supplemental material). In promastigotes, open chromatin frequency increases approximately 1,500 bp upstream of the first CDS of PTUs and remains high for the subsequent 5,000 bp (Fig. 3A). No such increase can be observed in the amastigote (Fig. 3B) and Pro/RAD (Fig. 3C) samples. In those, open chromatin is >5-fold less frequent both upstream and downstream of the PTU starts. This shows a clear preference for euchromatin near the expected start sites of polycistronic transcription in the logarithmically growing promastigotes and a prevalence of heterochromatin-like structures in the slowly proliferating amastigotes and RAD-treated promastigotes.

## DISCUSSION

We find that the differentiation from fast-growing promastigotes to slowly proliferating amastigotes is accompanied by a reduced accessibility of the genomic DNA for micrococcal nuclease, an effect that is commonly associated with the conversion from open euchromatin to condensed heterochromatin (30). This is not primarily due to the reduced growth of the differentiating cells, as a hydroxyurea-induced G_1_ growth arrest has no such effects (Fig. 1B). Rather, it appears as if triggers of life cycle stage differentiation, i.e., temperature, pH, and/or HSP90 inhibition, also trigger a reorganization of the nuclear chromatin.

More specifically, we observe the shift from Tn*5*-accessible euchromatin to inaccessible heterochromatin in the immediate upstream regions of most PTUs when L. donovani promastigotes are triggered to differentiate into amastigote-like *in vitro* forms. It must be noted, however, that a reverse change also appears at certain PTUs (see Fig. S3 in the supplemental material). It is not possible to link open or closed 5′ regions to specific gene functions since the proteins encoded within particular PTUs are not functionally linked (12).

Early work using *in vitro* chromosome condensation experiments at varying salt concentrations suggested a predominance of beads-on-a-string-structured 10-nm chromatin fibers and a lack of higher order 30-nm fibers in *Trypanosoma* (15). Yet, our results suggest a temperature-inducible condensation of chromatin in amastigote-like cells upstream of the PTUs where transcription is thought to initiate (14). Whether the compacted chromatin found in many dSSRs of amastigotes consists of condensed 30-nm fibers similar to those found in crown group eukaryotes, we cannot answer at this point.

The increased overall chromatin density observed by MNase digest assays and by our ATAC-seq analysis is compatible with our earlier finding that histone synthesis is greatly increased upon RAD-induced cellular differentiation (26) but seems to contradict earlier work, which showed increased histone-coding mRNA levels in the promastigote form (31–33). This can be explained, however, by the observed lack of correlation between mRNA levels and the synthesis and/or abundance of the encoded proteins in *Leishmania* (26, 34), a view that is supported by earlier comparative proteome analysis (20).

We observed a reduced general accessibility of the L. donovani chromatin under HSP90 inhibition, presumably caused by elevated histone synthesis (26), affecting the 5′ telomeric regions and the dSSRs, similar to what we observed in temperature/pH-induced axenic amastigotes (Fig. 2). Yet, the general chromosomal Tn*5* accessibility data (Fig. 2B; see Fig. S2 and S3 in the supplemental material) show that Pro/RAD chromatin outside of the dSSRs resembles that of promastigotes. It is noteworthy in this context that the HSP90 chaperone complex is well known to control chromatin architecture in eukaryotic model organisms (35, 36). We speculate that while HSP90 inhibition triggers a protein synthesis pattern (26, 37) similar to lege artis-produced (19) axenic amastigotes, it may also affect its ability to coordinate nucleosome assembly and/or positioning. Nevertheless, our results show that morphologically comparable axenic amastigotes and the RAD-induced amastigote-like cells (21, 22) have a similar chromatin density pattern inside transcription start regions.

When looking at cSSRs, we observe divergent Tn*5* accessibility, with more open chromatin in amastigotes. Earlier work by Lombraña et al. (28), using MNase-seq analysis, indicated a distinct drop of nucleosome occupancy in a small, ∼500-nt region downstream of transcription termination sites in L. major promastigote chromatin. In our ATAC analysis, we could not predict nucleosome positioning, only DNA accessibility, and we did not attempt to confirm these earlier results.

The presence of open chromatin in the rRNA-coding genes of trypanosomatids but also in the tRNA-coding cSSR regions has been described before using MNase digest assays (28, 29) and is validated by our ATAC-seq analysis (see Tables S2 and S3 in the supplemental material).

We attempted to correlate our ATAC-seq peak coverage with the results of an RNA-seq analysis (26) for promastigote cells, but we could not establish a correlation. RNA stability in *Leishmania* varies greatly between genes and developmental stages, confounding RNA-seq data. We therefore propose the future use of precision nuclear run-on sequencing (PRO-seq) analysis (38), a genome-wide nuclear run-on transcription assay, to correlate euchromatin prevalence with RNA-polymerase density across the genome. This methodology allows us to distinguish between active and paused transcription elongation complexes and should answer the underlying question of whether chromatin structure has an epigenetic impact on stage-specific transcription modulation.

Changes in the chromatin structure are also known to be regulated by posttranslational modifications (PTMs) of core histones, by the expression of histone variants, or due to DNA modifications. Fortunately, a host of work is already available in this context. For example, the acetylation of histone H3 in L. major was linked to polycistronic transcription origins and was ascribed an important regulatory role in transcription initiation. These acetylated histones were found mainly within dSSRs but also at telomeres and within PTUs (39). In addition, four histone acetyltransferases (HAT) were discovered in *Leishmania* spp. Using micrococcal nuclease digest assays, it was shown that overexpression of HAT2 increases accessibility of chromatin, while acetylation of histone H4 at Lys_4_ (H4K4) weakens interaction between neighboring nucleosomes (40). In another study, HAT2 was associated with cell cycle-specific activation of transcription start sites (41). An important DNA modification is base J (β-d-glucosyl-hydroxymethyluracil), which is necessary in L. major for transcription termination by RNA polymerase II, as readthrough transcription is observed in its absence (42). In T. cruzi, the absence of base J was also associated with H3/H4 acetylation, a reduction in the number of nucleosomes, and an accumulation of RNA Pol II at potential transcription starts (43). In parallel, the distribution of the variant histone H2AZ appears to be highly conserved among eukaryotes, and its incorporation into the chromatin of L. major affects the initiation of transcription (44). In T. brucei, the variant histones H2AZ and H2BV were localized at potential transcription start sites, whereas the variants H3V and H4V were found at termination sites (45).

Making use of histone gene deletions and/or histone point mutants at PTM sites, in conjunction with ATAC-seq analysis may give us a much-needed insight into the role played by epigenetic gene control in *Leishmania* and in other trypanosomatidic pathogens.

## MATERIALS AND METHODS

### Cell culture conditions for *Leishmania*.

Leishmania donovani 1S (MHOM/SD/62/1S) and L. donovani (HSP90rr) promastigotes were cultured as described in reference 26.

### Treatment of *Leishmania* with radicicol, hydroxyurea, and heat shock.

Logarithmically growing cells of L. donovani wild type (WT) or HSP90rr were adjusted to a cell density of 4 × 10^6^ cells/ml to a total volume of 10 ml and treated with 0, 200, 400, 800, or 1,600 ng/ml radicicol for the MNase digest assay. Incubation was carried out at 25°C for 72 h. For ATAC-seq, 1,200 ng/ml radicicol was used (IC_80_). Treatment with hydroxyurea was carried out on L. donovani WT cells at the same cell density with 0, 0.125, 0.1875, 0.25, 0.375, and 0.5 mM hydroxyurea and incubation at 25°C for 24 h. For heat shock, the cells were also adjusted to 4 × 10^6^ cells/ml to a total volume of 10 ml and incubated for 72 h at 37°C.

### Axenic amastigote differentiation.

Axenic amastigote differentiation was performed as previously described (46, 47). Briefly, mid-logarithmic-growth-phase (5 × 10^6^/ml) L. donovani promastigotes in modified medium 199 (20% fetal calf serum [FCS], pH 7.0) were shifted to 37°C for 24 h. Then, cells were precipitated (800 × *g*, 10 min, 20°C) and resuspended in medium 199 (20% FCS, pH 5.5). Incubation at 37°C with 5% CO_2_ was continued for 4 days, with fresh medium added after 2 days.

### Micrococcal nuclease digest.

The micrococcal nuclease assay was performed as already described for L. donovani (41). Cell concentration was determined using a CASY cell counter (Roche), and a total of 5 × 10^7^ cells were used per sample. Cells were washed once with 10 ml phosphate-buffered saline solution (pH 7) (48) before following the described protocol. For subsequent analysis, 3,000 ng of DNA per sample was applied to a 1.5% agarose gel (3 μg/ml ethidium bromide), and the intensity of the mononucleosome bands (λ = 305 nm) was quantified using ImageJ. The measured intensities were each normalized to the control of the same agarose gel.

### ATAC-seq.

ATAC-seq analysis was performed as described (49) with slight modifications. Briefly, 2 × 10^7^ cells were washed with 10 ml of cold phosphate-buffered saline solution (pH 7) and resuspended in 500 μl permeabilization buffer with 0.05% Triton X-100 (150 mM sucrose, 80 mM KCl, 35 mM HEPES, pH 7.4, 5 mM K_2_HPO_4_, and 5 mM MgCl_2_). Permeabilization was carried out for 5 min at room temperature before the samples were washed with permeabilization buffer without Triton X-100. For subsequent treatment with Tn*5* transposase, the pellet was resuspended in 50 μl transposition mix consisting of 25 μl 2× TD (reaction buffer from Nextera kit; Illumina) and 2.5 μl TDE1. As a control, 50 ng and 200 ng of L. donovani genomic DNA was treated with the transposition mix. Incubation was carried out at 37°C for 30 min, and the DNA was purified with Qiagen MinElute PCR purification kit (catalog no. 28004). Barcode PCR was optimized by quantitative PCR (qPCR) and performed using NEBNext high-fidelity 2× PCR master mix (M0541S) and Nextera XT index primer (FC-131-1001). The library was purified with AMPure XP beads (Beckman Coulter; A63880). Illumina sequencing was carried out according to the manufacturer’s instructions on a NextSeq 500/550 high output kit v2 (75 cycles) via paired-end sequencing.

### Bioinformatic analysis.

Reads (PRJNA739873) were trimmed and quality checked with Trimmomatic (50) and aligned to version 46 of the L. donovani BPK282A1 genome of TriTrypDB (51) using bowtie2 (52) with parameter X 2000. PCR duplicates were removed and pileups generated using SAMtools (53) before peak calling with Genrich (https://github.com/jsh58/Genrich). Peak length was calculated from the peak coordinates of the Genrich output.

### Data availability.

Reads can be accessed under BioProject accession number PRJNA739873.
