# Supplementary material for: Life Cycle Stage-Specific Accessibility of Leishmania donovani Chromatin at Transcription Start Regions
Source: mSystems. 2021 Jul 20;6(4):e00628-21. doi: 10.1128/mSystems.00628-21 (PMC8409730; doi:10.1128/mSystems.00628-21)
Supplement: TABLE S1 [file msystems.00628-21-st001.pdf]

Sample	Reads	Trimmed	Aligned
Pro	46861085	41341135	83.18%
	50964607	45292610	83.97%
Ama	101234567	91143536	43.42%
	56613102	50688537	45.67%
Pro/RAD	52507739	46491400	77.20%
	70559742	62331332	80.26%
gDNA control	53306328	46518293	95.04%
	59116619	51116634	94.81%

Table S1. ATAC-seq output. Two biological replicates each were used for sequencing of promastigotes (Pro), amastigotes (Ama), RAD-treated promastigotes (Pro/RAD) and gDNA controls. Numbers of total reads, number of reads after adapter trimming and percentage of reads aligned to the *L. donovani* reference genome are listed.
