# Supplementary material for: Life Cycle Stage-Specific Accessibility of Leishmania donovani Chromatin at Transcription Start Regions
Source: mSystems. 2021 Jul 20;6(4):e00628-21. doi: 10.1128/mSystems.00628-21 (PMC8409730; doi:10.1128/mSystems.00628-21)
Supplement: FIG S1 [file msystems.00628-21-sf001.pdf]

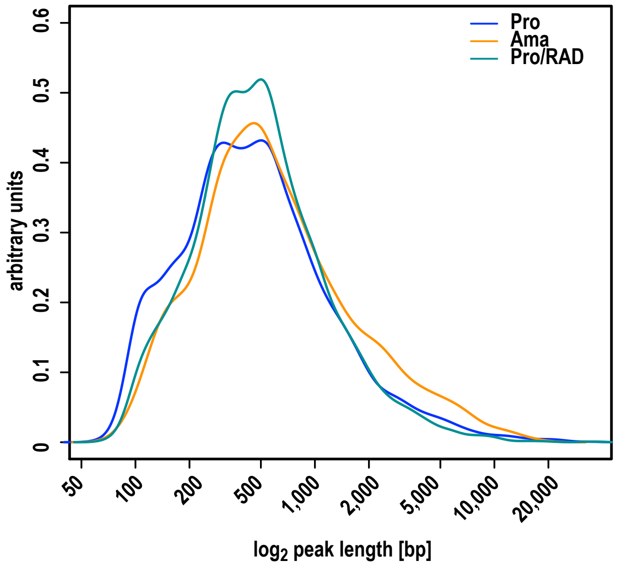

Figure S1. Frequency distribution of peak lengths. Peak lengths derived from Genrich peak calling were plotted against their relative frequencies for promastigotes (Pro, blue), axenic amastigotes (Ama, orange) and RAD-treated promastigotes (Pro/RAD, green).
