# Supplementary material for: Life Cycle Stage-Specific Accessibility of Leishmania donovani Chromatin at Transcription Start Regions
Source: mSystems. 2021 Jul 20;6(4):e00628-21. doi: 10.1128/mSystems.00628-21 (PMC8409730; doi:10.1128/mSystems.00628-21)
Supplement: FIG S2 [file msystems.00628-21-sf002.pdf]

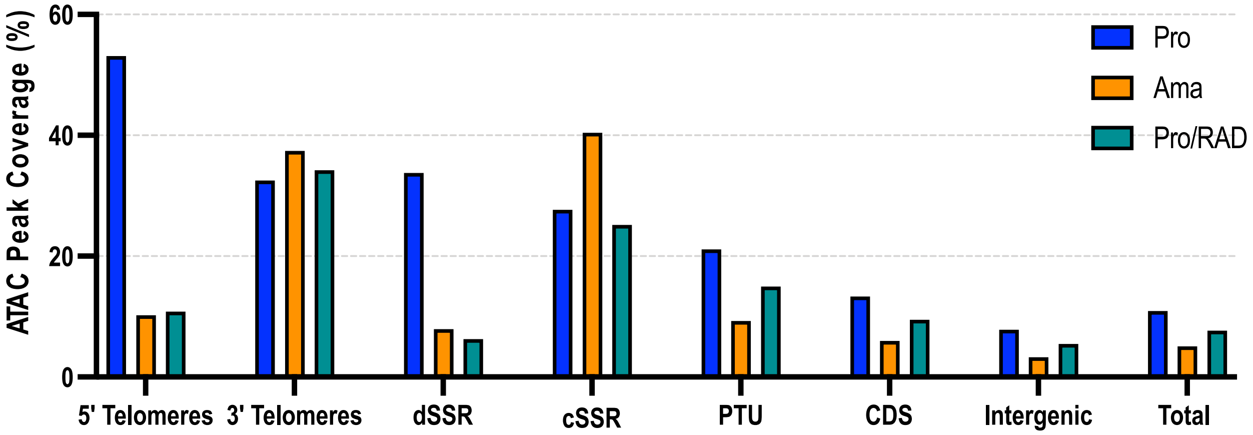

Figure S2. Distribution of open chromatin across the *L. donovani* genome. Coverage of ATAC peaks is indicated for promastigotes (Pro, blue), axenic amastigotes (Ama, orange) and RAD-treated promastigotes (Pro/RAD, green) at 5'- and 3'-telomeres, divergent (dSSRs) or convergent strand switch regions (cSSRs), polycistronic transcription units (PTUs), coding sequences (CDSs), intergenic regions within PTUs and total chromosomes. Data were pooled from 2 biological samples.
