# Supplementary material for: Life Cycle Stage-Specific Accessibility of Leishmania donovani Chromatin at Transcription Start Regions
Source: mSystems. 2021 Jul 20;6(4):e00628-21. doi: 10.1128/mSystems.00628-21 (PMC8409730; doi:10.1128/mSystems.00628-21)
Supplement: TABLE S2 [file msystems.00628-21-st002.pdf]

Table S2. ATAC peak coverage at tRNA genes.

Gene-ID	Lengths (bp)	ATAC Peaks (%) Pro	ATAC Peaks (%) Ama	ATAC Peaks (%) Pro/RAD
LdBPK_03tRNA1	73	100	100	100
LdBPK_05tRNA1	72	100	100	100
LdBPK_07tRNA1	62	100	100	100
LdBPK_09tRNA1	72	100	100	100
LdBPK_09tRNA2	83	100	100	100
LdBPK_09tRNA3	76	100	100	100
LdBPK_09tRNA4	71	100	100	100
LdBPK_09tRNA5	73	100	100	100
LdBPK_09tRNA6	72	100	100	100
LdBPK_09tRNA7	76	100	100	100
LdBPK_09tRNA8	72	100	100	100
LdBPK_09tRNA9	71	100	100	100
LdBPK_10tRNA1	73	100	100	100
LdBPK_10tRNA2	71	100	100	100
LdBPK_10tRNA3	73	100	100	100
LdBPK_11tRNA1	72	100	100	100
LdBPK_11tRNA2	73	100	100	100
LdBPK_11tRNA3	72	100	100	100
LdBPK_161tRNA1	72	100	100	100
LdBPK_17tRNA1	72	100	100	100
LdBPK_17tRNA2	81	100	100	100
LdBPK_17tRNA3	73	100	100	100
LdBPK_21tRNA1	81	100	100	100
LdBPK_21tRNA2	72	100	100	100
LdBPK_21tRNA3	74	100	100	100
LdBPK_21tRNA4	73	100	100	100
LdBPK_23tRNA1	74	100	100	100
LdBPK_23tRNA10	72	100	100	100
LdBPK_23tRNA2	73	100	100	100
LdBPK_23tRNA3	72	100	100	100
LdBPK_23tRNA4	72	100	100	100
LdBPK_23tRNA5	83	100	100	100
LdBPK_23tRNA6	72	100	100	100
LdBPK_23tRNA7	72	100	100	100

Table S2. ATAC peak coverage at tRNA genes.

Gene-ID	Lengths (bp)	ATAC Peaks (%) Pro	ATAC Peaks (%) Ama	ATAC Peaks (%) Pro/RAD
LdBPK_23tRNA8	73	100	100	100
LdBPK_23tRNA9	72	100	100	100
LdBPK_24tRNA1	72	100	100	100
LdBPK_24tRNA2	72	100	100	100
LdBPK_24tRNA3	82	100	100	100
LdBPK_24tRNA4	136	100	100	100
LdBPK_24tRNA5	72	100	100	100
LdBPK_29tRNA1	82	100	100	100
LdBPK_29tRNA2	81	100	100	100
LdBPK_30tRNA1	72	100	100	100
LdBPK_31_tRNA1	73	100	100	100
LdBPK_31_tRNA2	71	100	100	100
LdBPK_31_tRNA3	72	100	100	100
LdBPK_31_tRNA4	73	100	100	100
LdBPK_31_tRNA5	81	100	100	100
LdBPK_33tRNA1	73	100	100	100
LdBPK_33tRNA2	73	100	100	100
LdBPK_33tRNA3	72	100	100	100
LdBPK_34tRNA1	74	100	100	100
LdBPK_34tRNA2	74	100	100	100
LdBPK_34tRNA3	73	100	100	100
LdBPK_34tRNA4	80	100	100	100
LdBPK_34tRNA5	81	100	100	100
LdBPK_34tRNA6	7	100	100	100
LdBPK_36tRNA1	72	100	100	100
LdBPK_36tRNA2	72	100	100	100
LdBPK_36tRNA3	72	100	100	100
LdBPK_36tRNA4	82	100	100	100
LdBPK_36tRNA5	72	100	100	100
LdBPK_36tRNA6	72	100	100	100
