# Supplementary material for: Life Cycle Stage-Specific Accessibility of Leishmania donovani Chromatin at Transcription Start Regions
Source: mSystems. 2021 Jul 20;6(4):e00628-21. doi: 10.1128/mSystems.00628-21 (PMC8409730; doi:10.1128/mSystems.00628-21)
Supplement: TABLE S3 [file msystems.00628-21-st003.pdf]

Table S3. ATAC peak coverage at rRNA genes.

Gene-ID	Lengths (bp)	ATAC Peaks (%) Pro	ATAC Peaks (%) Ama	ATAC Peaks (%) Pro/RAD
LdBPK_05rRNA2	81	100	100	100
LdBPK_09rRNA1	116	100	100	100
LdBPK_09rRNA2	68	100	100	100
LdBPK_09rRNA3	303	100	100	100
LdBPK_11rRNA1	99	100	100	77
LdBPK_21rRNA1	231	100	100	100
LdBPK_23rRNA1	120	100	100	100
LdBPK_27rRNA3	213	100	100	100
LdBPK_27rRNA4	1782	100	100	100
LdBPK_27rRNA5	262	100	100	100
LdBPK_27rRNA6	1549	100	100	100
