# Supplementary material for: Life Cycle Stage-Specific Accessibility of Leishmania donovani Chromatin at Transcription Start Regions
Source: mSystems. 2021 Jul 20;6(4):e00628-21. doi: 10.1128/mSystems.00628-21 (PMC8409730; doi:10.1128/mSystems.00628-21)

Figure S3. Genrich peak calling for all 36 chromosomes in *L. donovani*. Peak calling was performed for two biological repeats and is depicted for promastigotes (Pro, blue), axenic amastigotes (Ama, orange) and RAD-treated promastigotes (Pro/RAD, green). Coding sequences (CDS) in two different orientations are indicated with black dots (forward direction) and red dots (reverse direction).

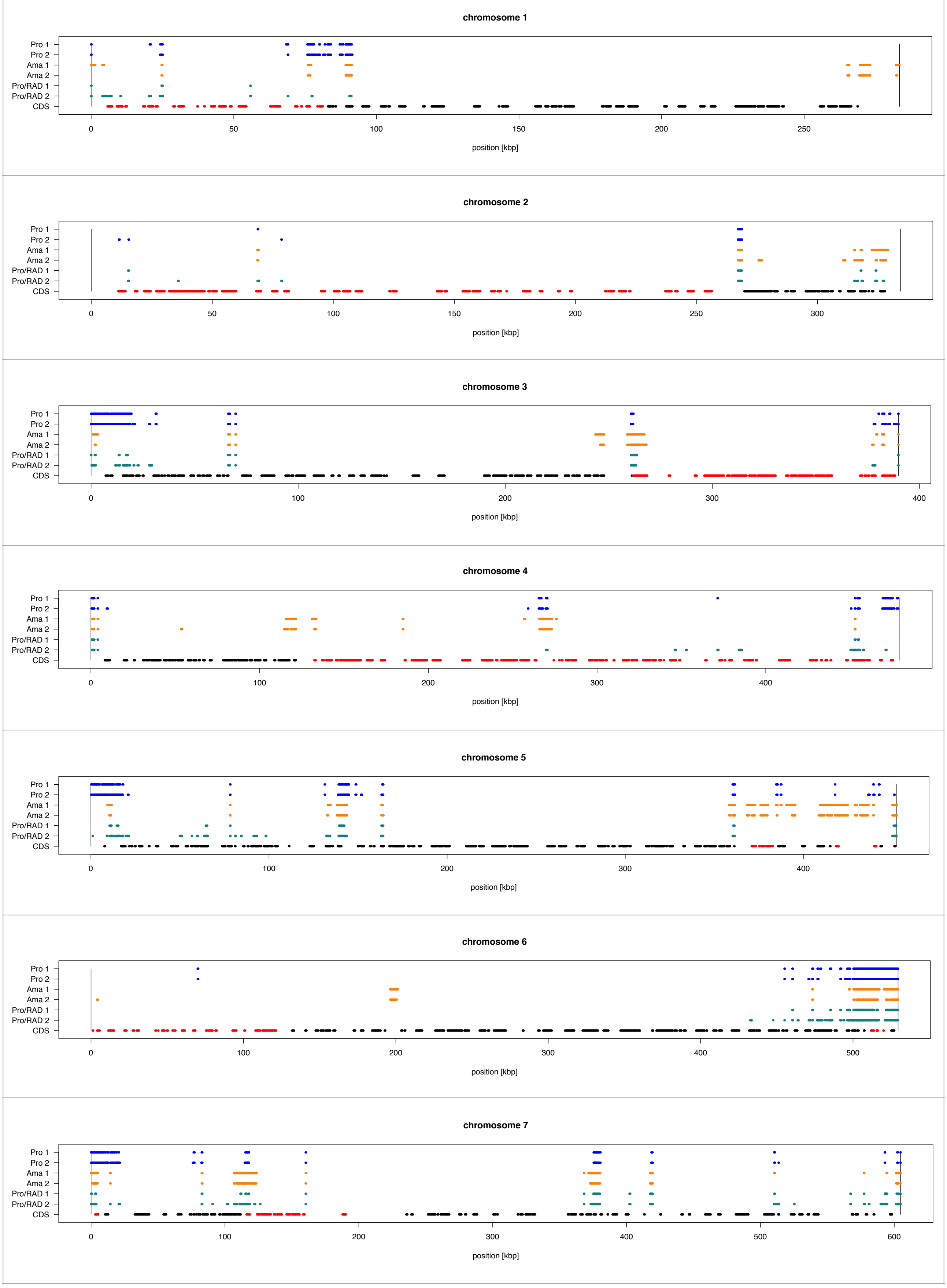

Figure S3. Genrich peak calling for all 36 chromosomes in *L. donovani*. Peak calling was performed for two biological repeats and is depicted for promastigotes (Pro, blue), axenic amastigotes (Ama, orange) and RAD-treated promastigotes (Pro/RAD, green). Coding sequences (CDS) in two different orientations are indicated with black dots (forward direction) and red dots (reverse direction).

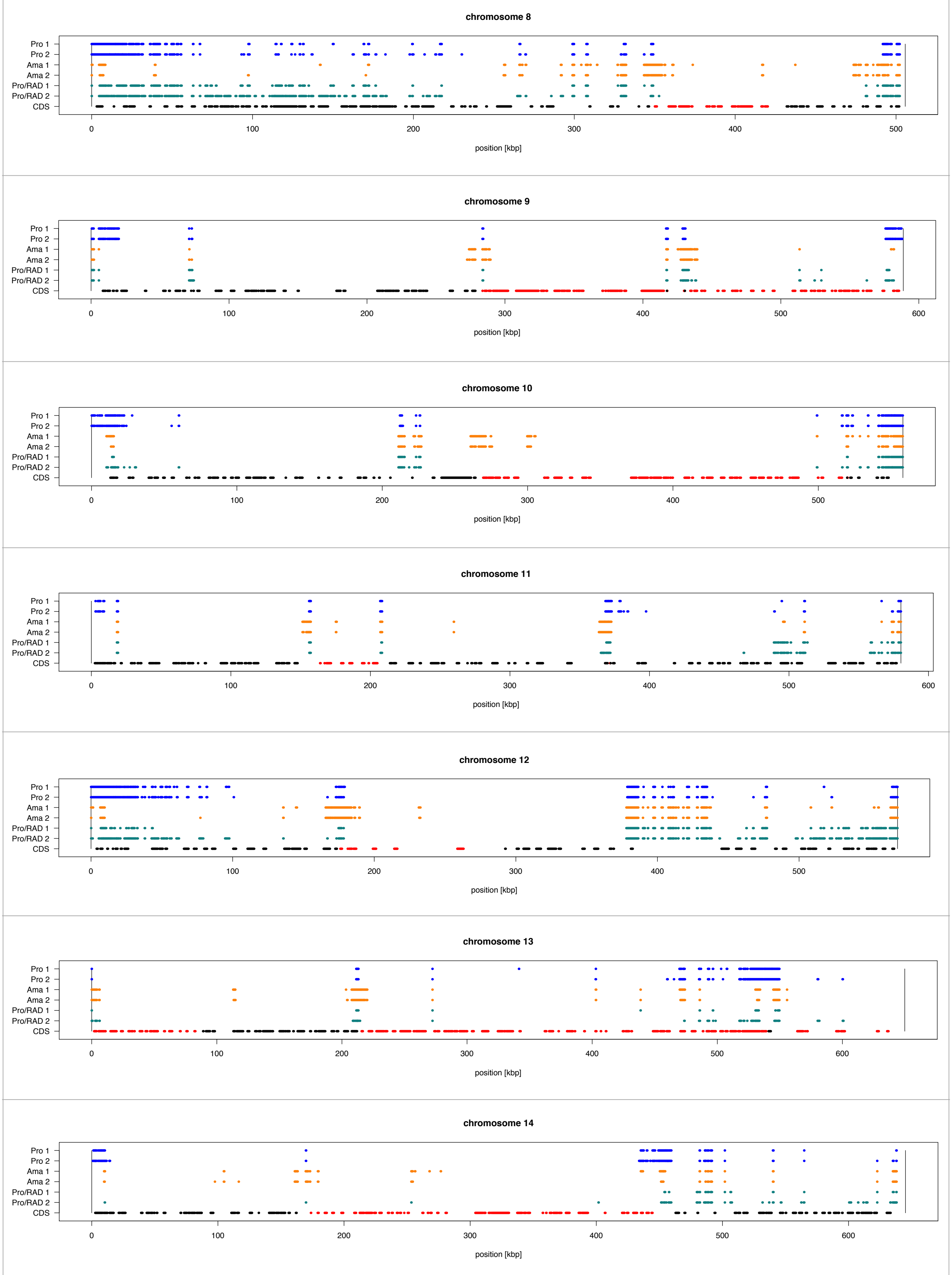

Figure S3. Genrich peak calling for all 36 chromosomes in *L. donovani*. Peak calling was performed for two biological repeats and is depicted for promastigotes (Pro, blue), axenic amastigotes (Ama, orange) and RAD-treated promastigotes (Pro/RAD, green). Coding sequences (CDS) in two different orientations are indicated with black dots (forward direction) and red dots (reverse direction).

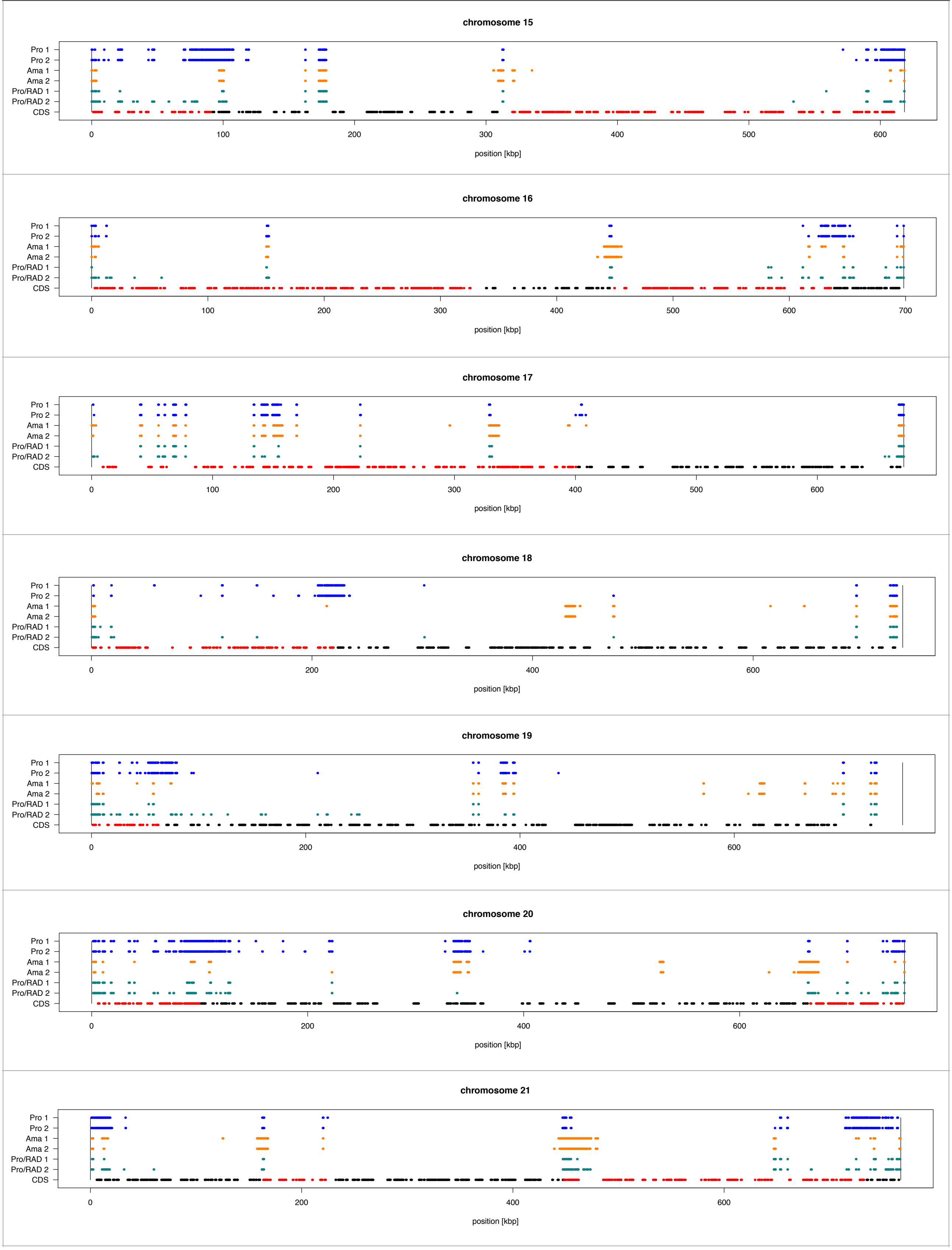

Figure S3. Genrich peak calling for all 36 chromosomes in *L. donovani*. Peak calling was performed for two biological repeats and is depicted for promastigotes (Pro, blue), axenic amastigotes (Ama, orange) and RAD-treated promastigotes (Pro/RAD, green). Coding sequences (CDS) in two different orientations are indicated with black dots (forward direction) and red dots (reverse direction).

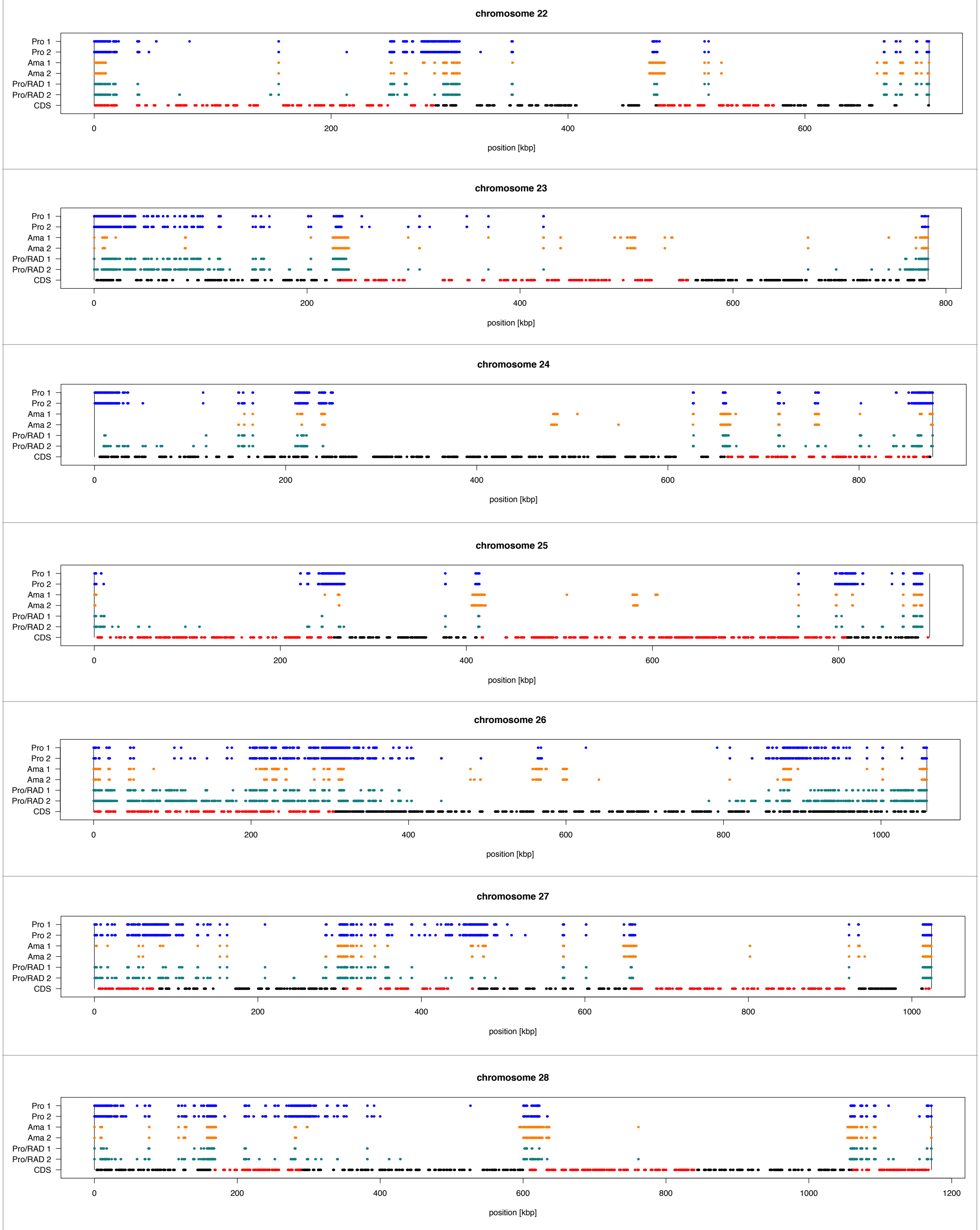

Figure S3. Genrich peak calling for all 36 chromosomes in *L. donovani*. Peak calling was performed for two biological repeats and is depicted for promastigotes (Pro, blue), axenic amastigotes (Ama, orange) and RAD-treated promastigotes (Pro/RAD, green). Coding sequences (CDS) in two different orientations are indicated with black dots (forward direction) and red dots (reverse direction).

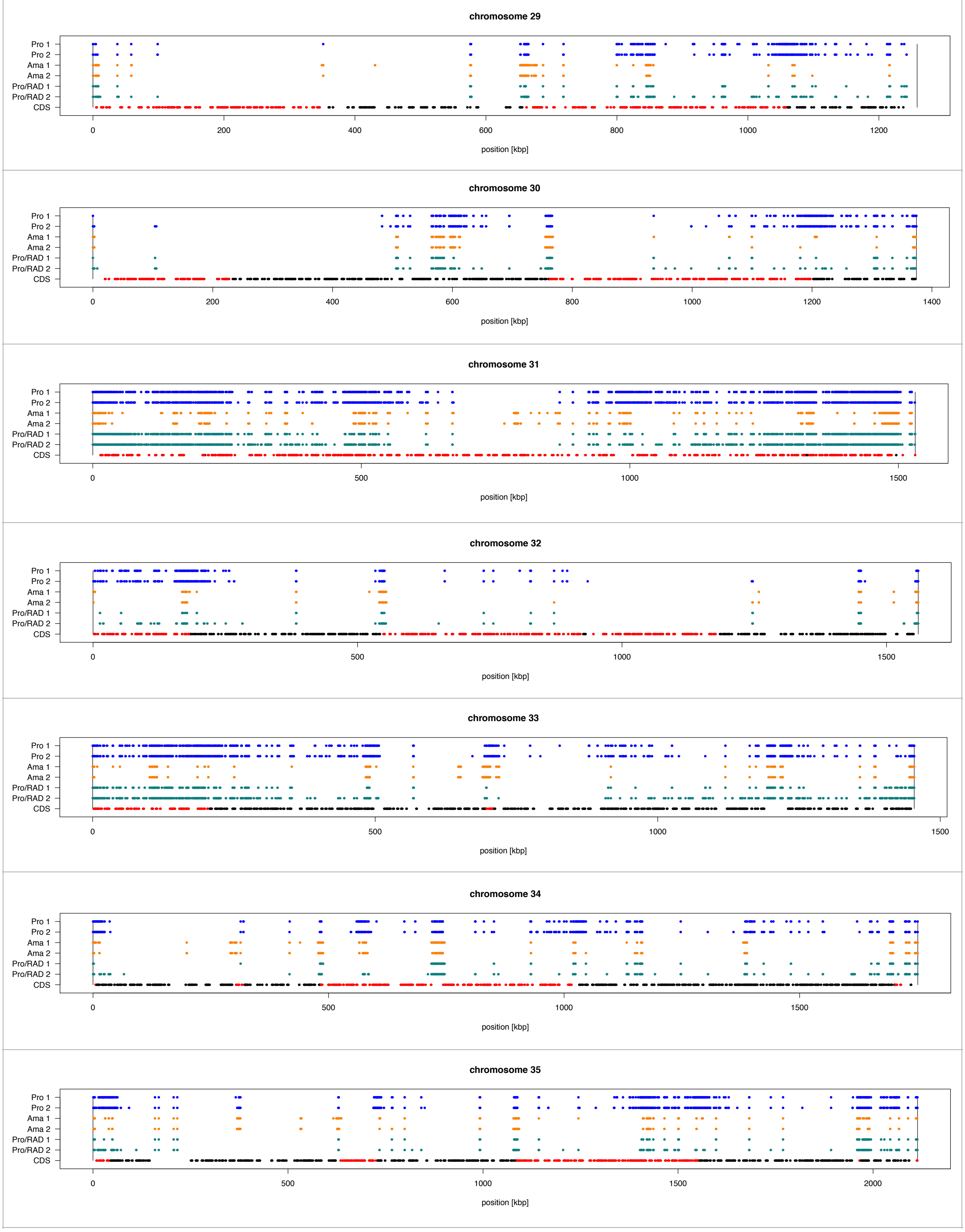

Figure S3. Genrich peak calling for all 36 chromosomes in *L. donovani*. Peak calling was performed for two biological repeats and is depicted for promastigotes (Pro, blue), axenic amastigotes (Ama, orange) and RAD-treated promastigotes (Pro/RAD, green). Coding sequences (CDS) in two different orientations are indicated with black dots (forward direction) and red dots (reverse direction).

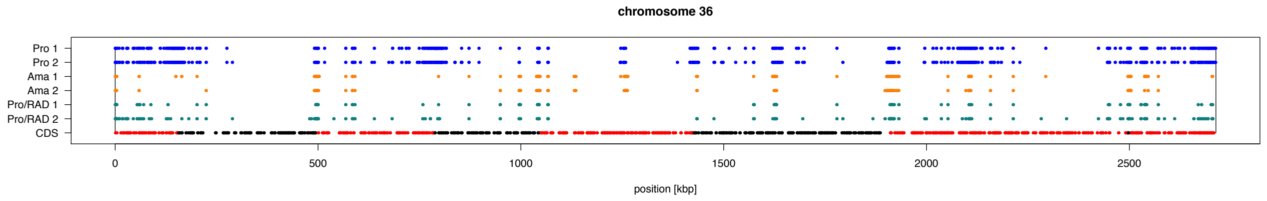

Supplement: FIG S3 [file msystems.00628-21-sf003.pdf]
